# Surveillance of Emerging Rodent-Borne Pathogens in Wastewater in Taiwan: A One Health Approach

**DOI:** 10.3390/tropicalmed9110282

**Published:** 2024-11-18

**Authors:** Kun-Hsien Tsai, Tsai-Ying Yen, Hsin-Hsin Tung, Amy Ho, Yang-Ta Chien, Chung-Yu Wang, Shu-Wei Kang, Ning-Ning Juan, Fang-Ling Lin

**Affiliations:** 1 Institute of Environmental and Occupational Health Sciences, College of Public Health, National Taiwan University, Taipei 100025, Taiwan; amyho1997@gmail.com (A.H.); r11852029@ntu.edu.tw (Y.-T.C.); frhlqp@gmail.com (C.-Y.W.); swkang@ntu.edu.tw (S.-W.K.); r13852012@ntu.edu.tw (N.-N.J.); d12852007@ntu.edu.tw (F.-L.L.); 2 Global Health Program, College of Public Health, National Taiwan University, Taipei 100025, Taiwan; 3 Center for Diagnostics and Vaccine Development, Centers for Disease Control, Ministry of Health and Welfare, Taipei 115201, Taiwan; 4 Graduate Institute of Environmental Engineering, National Taiwan University, Taipei 106319, Taiwan; htung@ntu.edu.tw

**Keywords:** wastewater-based epidemiology, *Leptospira interrogans*, hantaviruses, RHEV, *Rattus norvegicus*, One Health

## Abstract

Leptospirosis and hantavirus syndrome are two major rodent-borne diseases in Taiwan. *Rocahepevirus ratii* (RHEV), a virus closely related to hepatitis E virus (HEV, *Paslahepevirus balayani*), is emerging and has been reported to cause hepatitis in humans. We employed wastewater-based epidemiology to actively monitor rodent-borne pathogens, and the correlations with human cases were evaluated. Wastewater was collected using grab sampling at 11 sites along a sewer system including influents and effluents at a wastewater treatment plant in Tamsui, New Taipei City, Taiwan, monthly during June 2023 to May 2024. The presence of pathogens was examined by reverse transcription-polymerase chain reaction (RT-PCR). The result showed an overall positivity rate of 38.2% (50/131). *Leptospira* was detected most often (48/131, 36.6%), and RHEV and hantaviruses were found once each during the study period. Sequencing identified *Leptospira interrogans* close to isolates from rodents and human cases, while sequences of hantavirus and RHEV were most similar to isolates from rodents. No significant correlation was found with human cases or positive samples for rodent DNA. Here, we present an example of a One Health approach applying wastewater to environmental surveillance for the early detection and prevention of emerging diseases.

## 1. Introduction

Wastewater-based epidemiology has long been used for disease surveillance [1,2,3]. It received increased attention during the COVID-19 pandemic, especially when studies have shown that SARS-CoV-2 was not only detectable in wastewater, but that detection of viral RNA could reflect or even forewarn the occurrence of outbreaks [4,5,6]. Further research also demonstrated the feasibility of identifying SARS-CoV-2 variants in wastewater [7]. The concept of wastewater-based epidemiology is that sewage collects pathogens in wash-off or body fluids excreted by infected individuals, such as urine, sweat, saliva, blood, and feces, and therefore, examination of pathogens in sewage would reveal infections, and moreover, epidemic dynamics in communities. Compared to clinical cases, wastewater employs environmental specimens and provides a tool to monitor pathogens circulating in communities with few samples.

According to the World Health Organization, 75% of new human pathogens detected in the past three decades have originated from animals, and around 60% of emerging infectious diseases are zoonoses [8]. Therefore, early detection and prevention of emerging disease outbreaks require a One Health approach integrating multi-disciplinary efforts to protect human, animal, and environmental health. Besides medicine and veterinary medicine, environmental science applying wastewater to the surveillance of potential pathogens offers an opportunity to discover new zoonotic infections as well as assess epidemic risks promptly and cost-effectively [9,10]. Take mpox as an example. A global outbreak of mpox started in 2022, and the utility of wastewater-based epidemiology for mpox virus (MPXV) was explored [11,12,13]. Not only a positive correlation between MPXV DNA concentrations in wastewater and the incidence was observed, but MPXV DNA was detected prior to reported cases [14,15,16]. In addition, a recent report testing influenza A virus and H5 subtype showed a high correlation between reported infected herds of dairy cattle, poultry, or other animals and the detection of highly pathogenic avian influenza (HPAI) A (H5N1) virus in wastewater [17]. Nevertheless, wastewater surveillance could be particularly useful in tracking viruses that cause human infection resulting in nonspecific, mild, or no symptoms. In some European countries, hepatitis E virus (HEV, *Paslahepevirus balayani*) and rat hepatitis E virus (RHEV, *Rocahepevirus ratii*) were often recognized in wastewater, although relatively few human clinical cases were reported [18,19,20,21,22]. Wastewater-based epidemiology has also been proposed as alternatives for the surveillance of vector-borne viruses such as Zika virus, dengue virus, and chikungunya virus [23,24]. In this study, we present an example of monitoring humans and adjacent environments by screening for rodent-borne zoonoses using a wastewater-based One Health approach in an urban setting.

Leptospirosis and hantavirus syndrome are two major rodent-borne infectious diseases in Taiwan, and they caused 950 and 45 cases during January 2014 to August 2024, respectively [25]. Leptospirosis is caused by infection of *Leptospira interrogans* sensu lato, a Gram-negative spirochete belonging to the family Leptospiraceae. Twenty-five serogroups containing more than 200 serotypes of *L. interrogans* sensu lato have been identified in patients. The most common serotype in Taiwan is *Shermani* (*Leptospira santarosai* serovar *Shermani*) [26]. *Leptospira* can infect a variety of animals. Rodents are the primary carriers of leptospirosis, transmitting the pathogen through urine-contaminated water. Humans contract the spirochete through mucous membranes and skin and by the consumption of contaminated water. Clinical symptoms can range from a headache, muscle pains, and fevers to jaundice, kidney failure, bleeding in lungs, and meningitis. In Taiwan, epidemics occurred during May to October when heavy rains in plum rain season or typhoons led to floods [26,27].

Hantavirus syndrome such as hantavirus hemorrhagic fever with renal syndrome (HFR) and hantavirus pulmonary syndrome (HPS) is caused by infection of hantaviruses, also known as orthohantaviruses, belonging to a genus of negative-sense single-stranded RNA viruses in the family *Hantaviridae* within the order *Bunyavirales*. Humans become infected through contact with contaminated rodent urine, saliva, and feces. Inhalation of aerosolized rodent excreta containing virus particles may lead to HPS. Symptoms of hantavirus syndrome include fatigue, a headache, fevers, abdominal pains, hypotension, thrombocytopenia, leukocytosis, renal dysfunction, and respiratory illness. The infection could be severe and deadly. Cases of hantavirus syndrome occurred sporadically in Taiwan although evidence of rodent infections has been found in ports around the islands [28]. Studies have observed a seroprevalence of 6.0–13.0% in rodent-shaped small mammals. The seropositivity rates were 11.5–19.1%, 1.0–2.6%, 1.0–6.4%, 0.25–5.3%, and 0–7.7% for *Rattus norvegicus*, *Rattus tanezumi*, *Suncus murinus*, *Rattus losea*, and *Mus musculus*, respectively [28,29,30,31].

Hepatitis E is an infection caused by HEV (*P. balayani*), a member of the *Hepeviridae* family with a positive-sense single-stranded RNA genome. Four genotypes of HEV are associated with human infections. While genotype 1 and 2 spread mainly through fecal-oral routes and lead to outbreaks in Southeast Asia, India, Africa, and Central America, genotype 3 and 4 circulate in animals and occasionally infect humans [32,33]. Hepatitis E is generally a self-limiting illness, but it can be severe in pregnant women and immunosuppressed individuals. The fatality rate can reach 20% in pregnant women developing fulminant liver failure [33]. The disease occurred sporadically in Taiwan, with 99 confirmed cases diagnosed during 2014 to 2023 [25]. A study using the recombinant ORF2 protein as an antigen showed that 8.9% (112/1250) of the study subjects had anti-HEV antibodies [34]. Recently, another species of hepeviruses, *R. ratii* (rat HEV, RHEV), has been reported causing a new zoonotic disease resulting in acute and chronic hepatitis in humans [35]. The virus was first identified in Germany in 2010 and has been detected in rodents in the United States, Canada, China, Vietnam, and Europe [36,37]. Human cases of RHEV infection have been documented in Hong Kong, Canada, Spain, France, and Japan [35,38,39,40,41,42,43]. In Taiwan, the presence of RHEV infection in rodents was supported by the detection of viral RNA in 4% (2/50) and antibodies in 52% (26/50) of the rodent sera collected near ports. However, screening sera from patients suspected with acute hepatitis E did not find RHEV infection [44].

Examinations of reservoirs and hosts for infections have been used to assess risks of diseases. However, sampling usually involves capture of animals, which either disturbs the ecology, or uses procedures stressful for the animals, putting researchers at risk of infections. Moreover, capture rates may vary, and results may not be representative. Wastewater collecting excreta from humans and synanthropic animals could give an overview of potential pathogens circulating in the area. In the study, we aimed to employ wastewater-based epidemiology to actively monitor rodent-borne zoonoses including leptospirosis, hantavirus syndrome, and HEV/RHEV infections in a district of the Taipei Metropolitan Area, and the correlations with human cases during the study period were evaluated.

## 2. Materials and Methods

### 2.1. Study Site

**Figure 1:**
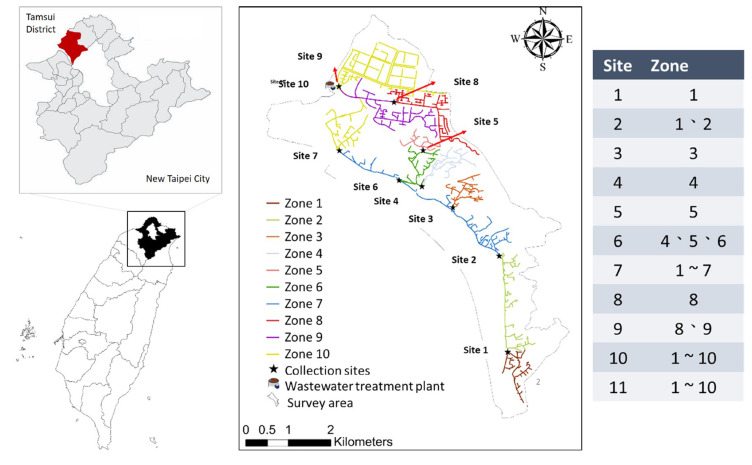
The sampling sites of wastewater in Tamsui, New Taipei City, Taiwan.

Tamsui is a district in New Taipei City in northern Taiwan. It is located on the north bank of the lower reaches of Tamsui River, facing the Taiwan Strait to the northwest. Historically, it was the starting point for Spain to land in Taiwan and establish a maritime trade relay base. After the 1990s, driven by the Danhai New Township Urban Plan, various transportation constructions were gradually established, making Tamsui a popular district of the Taipei Metropolitan Area with growing populations. The Tamsui sewer system serves 61,560 households, comprising 96.6% of households in the area. In the study, 11 sampling sites along the Tamsui sewer system were selected. Site 1 was located right next to a hospital, and Site 10 and 11 were at the inlet and outlet points of the wastewater treatment plant (Figure 1). The downstream relationships of the sampling sites and the zones covered by each sampling site were illustrated. The source of wastewater was mainly domestic sewage.

### 2.2. Sampling

Wastewater was collected through the manholes at Site 1 to 9 monthly during June 2023 to May 2024. The Tamsui Wastewater Treatment Plant employs a three-stage purification including physical separation, activated sludge, and sand filtration followed by UV disinfection to treat sewage before discharging it to the estuary of Tamsui River. Raw influent samples and post-treated effluent samples were also collected monthly at Site 10 from June to May 2024 (*n* = 12) and at Site 11 from July 2023 to May 2024 (*n* = 11), respectively. The sampling frequency was determined by taking into account the relatively low number of cases caused by the target pathogens, persistence of pathogens in reservoirs, sampling method used, and availability of the workforce. Grab sampling was applied to the study. Briefly, wastewater was randomly sampled using a 20 L bucket at each site once a month and placed into three 1000 mL sterile plastic jars. The jars, one for characteristic measurements and two for pathogen detection, were transported back to our laboratory on ice.

### 2.3. Measurement of Wastewater Quality Indicators

Temperature, pH, and dissolved oxygen of wastewater samples were recorded on-site immediately after sampling using a portable DO/pH/conductivity kit (Extech Instruments Corporation, Nashua, NH, USA), while biochemical oxygen demand (BOD), chemical oxygen demand (COD), ammonia nitrogen (NH_3_-N), total nitrogen, and total phosphate were measured by a commercial laboratory (Precica Environment Coporation, Taipei, Taiwan). In principle, BOD was determined by the dilution method, which mixes the samples with buffered dilution water dosed with seed microorganisms and calculates the amount of dissolved oxygen consumed after five days of incubation in a dark room at 20 °C. COD was evaluated using potassium dichromate and titrated with ferrous ammonium sulfate until the ferroin indicator changed from blue-green to a reddish brown color. NH_3_-N was analyzed using the phenate method, and the absorbance of a 640 nm wavelength was measured. Total nitrogen was the sum of the nitrate-nitrogen (NO_3_-N), nitrite-nitrogen (NO_2_-N), and the Kjeldahl nitrogen. Nitrate-nitrogen in the samples was determined by ion chromatography, while nitrite-nitrogen was determined through the formation of a reddish purple azo dye produced by coupling diazotized sulfanilamide with N-(1-naphthyl)-ethylenediamine dihydrochloride (NED dihydrochloride) under acidic conditions. The Kjeldahl analysis consisted of digestion, distillation, and titration, which subsequently converted organic nitrogen into ammonium, ammonia, and ammonium sulfate. Total phosphate was assessed by the ascorbic acid-Molybdenum method. All the analyses were carried out in accordance with the protocols of the Ministry of Environment, Taiwan.

### 2.4. RNA Extraction

Total RNA was purified using the RNeasy^®^ PowerFecal^®^ Pro Kit (Qiagen, Hilden, Germany) following the manufacturer’s instructions. Ninety milliliters of wastewater from each jar were used for RNA extraction. Briefly, the samples were centrifuged at 4500× *g* at 4 °C for 2 h. The precipitates were re-suspended in a mixture of 650 μL CD1 and 100 μL Trizol solutions and added to PowerBead Pro Tubes and homogenized. After centrifugation, the supernatant was mixed with a CD2 solution to remove inhibitors. The remaining stool particles were centrifuged and discarded, and the supernatant was mixed with ethanol. RNA purification was performed using MB RNA Spin Columns with on-column Dnase digestion. A volume of 100 μL nuclease-free water was used for elution. Purified RNA was either used immediately for reverse transcription (RT) or stored at −80 °C for up to one week.

### 2.5. Molecular Analysis

First-strand cDNA was synthesized using SuperScript^®^ IV Reverse Transcriptase (Thermo Fisher Scientific Inc., Waltham, MA, USA). PCR was carried out with the mixture of 2 μL of template cDNA, 1 μL of 10 μM forward primer, 1 μL of 10 μM reverse primer, 0.9 μL 50 mM MgCl_2_, 2.5 μL 10× PCR Buffer, 0.5 μL 10 mM dNTP mix, and 0.5 μL Platinum^TM^ Taq DNA Polymerase (Thermo Fisher Scientific Inc., Waltham, MA, USA), and a final volume of 25 μL was obtained with ddH_2_O. The reaction conditions included an initial incubation of 95 °C for 15 min, 35 cycles of denaturation at 94 °C for 30 s, annealing at a temperature depending on the primers used (Table S1) for 30 s, and extension at 72 °C for 40 s, followed by a final extension at 72 °C for 10 min and a 16 °C hold [45,46,47,48]. The PCR products were visualized on 2% agarose gels stained with EZ-Vision^®^ DNA Dye (Amresco Inc., Solon, OH, USA). The positive amplicons were sent for bidirectional sequencing at a commercial laboratory (Mission Biotech, Taipei, Taiwan) where Sanger sequencing was performed using an ABI PRISM^®^ 3730XL DNA Analyzer (Applied Biosystems, Waltham, MA, USA) [49]. Raw sequences were assembled and checked by SeqMan (DNASTAR, Madison, WI, USA) [50]. The obtained sequences were compared with those available in GenBank using BLAST. Sequences were then aligned using the Clustal W algorithm implemented in BioEdit v7.2.5 software [51]. The resulting alignment was analyzed using MEGA11 [52]. Two replicates of results were yielded for each sample (sampling point), and the sample was considered positive if any of the replicates were positive.

### 2.6. Statistics

Human case numbers were obtained from the Taiwan National Infectious Disease Statistics System (NIDSS) [9]. Seasonal differences in variables were compared using ANOVA. By definition, spring starts in March and lasts to May; summer starts in June and lasts to August; autumn is from September to November; and winter is from December to February in Taiwan. Correlations between monthly positivity rates of rodent-borne pathogens detected and numbers of human cases or positive samples for rodent DNA were analyzed using Pearson’s correlations as an indicator of the strength of linear relationships within the dataset. The data were tabulated in Excel, and the analyses were performed in IBM SPSS version 26.0 software (SPSS Inc., Chicago, IL, USA).

## 3. Results

### 3.1. Wastewater Samples

A total of 131 wastewater samples were collected. The average temperature of water was 25.0 ± 3.1 °C, with the lowest temperature of 20.7 ± 1.0 °C observed in January 2024 and the highest temperature of 28.5 ± 1.3 °C in July 2023 (Table 1). The average pH value was 8.0 ± 0.5, ranging from 6.07 to 9.07. The BOD, COD, NH_3_-N, total nitrogen, and total phosphate in wastewater samples all met the quality standards for discharge into the sewer system stipulated by the local government, which require BOD, COD, NH_3_-N, total nitrogen, and total phosphate to be ≤ 450, 600, 75, 90, and 20 mg/l, respectively.

### 3.2. Detection of Leptospira spp. in Wastewater and Human Cases with Leptospirosis

**Figure 2:**
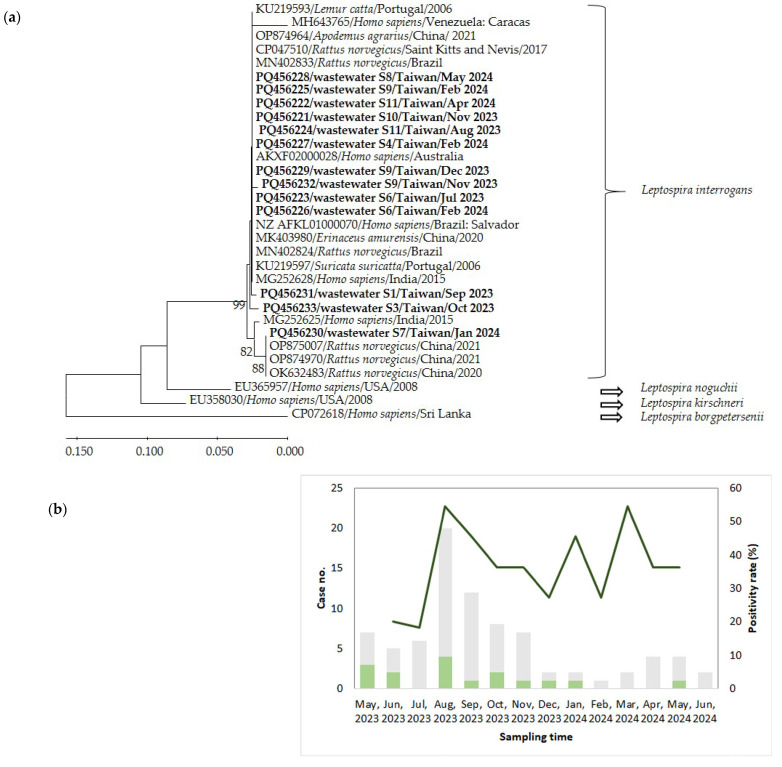
(**a**) The phylogenetic analysis of *Leptospira* spp. based on the partial nucleotide sequence of secY (245 nt). *Leptospira borgpetersenii* was used for the outgroup species. The evolutionary relationships were inferred by a neighbor-joining method with 1000 bootstrap replicates. Sequences obtained in the study were written in bold, and the sampling sites and time were indicated. (**b**) The monthly positivity rates of *Leptospira* detected in wastewater and numbers of leptospirosis cases.

*Leptospira* cDNA was detected in 48 samples including two effluent samples at Site 11, which contained treated wastewater at the end of pipelines in the Tamsui Wastewater Treatment Plant, resulting in an overall detection rate of 36.6% (48/131) (Table 2). *Leptospira* spp. were found most frequently at Site 6 (8 of 12 months, 66.7%), which contained wastewater from Zone 4, 5, and 6, and least frequently at Site 8 (1 of 12 months, 8.3%). The positivity rates in each month ranged from 18.2% (2 of 11 sites) in July 2023 to 54.5% (6 of 11 sites) in August 2023 and March 2024, with the pathogen detectable throughout the year. The positivity rates were not significantly different between seasons (*F* = 0.5, *p* = 0.688). Sequencing of the PCR products identified *L. interrogans* in wastewater (Figure 2a). Five different sequences were obtained. Sequences of amplicons from wastewater collected at Site 6 in July 2023 (PQ456223), Site 11 in August 2023 (PQ456224), Site 10 in November 2023 (PQ456221), Site 9 in December 2023 (PQ456229), Site 4 in February 2024 (PQ456227), Site 6 in February 2024 (PQ456226), Site 9 in February (PQ456225), Site 11 in April 2024 (PQ456222), and Site 8 in May 2024 (PQ456228) were identical to each other and to reference sequences from *R. norvegicus* (MN402833, CP047510, and CP047510) and patients (NZ AFKL01000070 and KXF02000028). The amplicon sequences from wastewater collected at Site 9 in November (PQ456232), Site 1 in September (PQ456231), and Site 3 in October (PQ456233) in 2023 were 99.6, 99.6, and 99.18% similar to the above amplicons and the sequence from *R. norvegicus* (MN402833), respectively. The sequence of the sample collected at Site 7 in January 2024 (PQ456230) was identical to reference sequences from *R. norvegicus* in China (OP874970 and OK632483).

According to the Taiwan Centers for Disease Control (Taiwan CDC), 73 confirmed cases of leptospirosis were diagnosed during the study period, 13 of which were located in the Taipei Region, and most cases occurred in August 2023. The seasonal difference in case numbers in the Taipei Region was not significant (*F* = 1.3, *p* = 0.336). We did not observe significant correlation between the number of cases and the monthly positivity rates (r = −0.372, *p* = 0.233) (Figure 2b).

### 3.3. Detection of Hantaviruses in Wastewater and Human Cases with Hantavirus Syndrome

**Figure 3:**
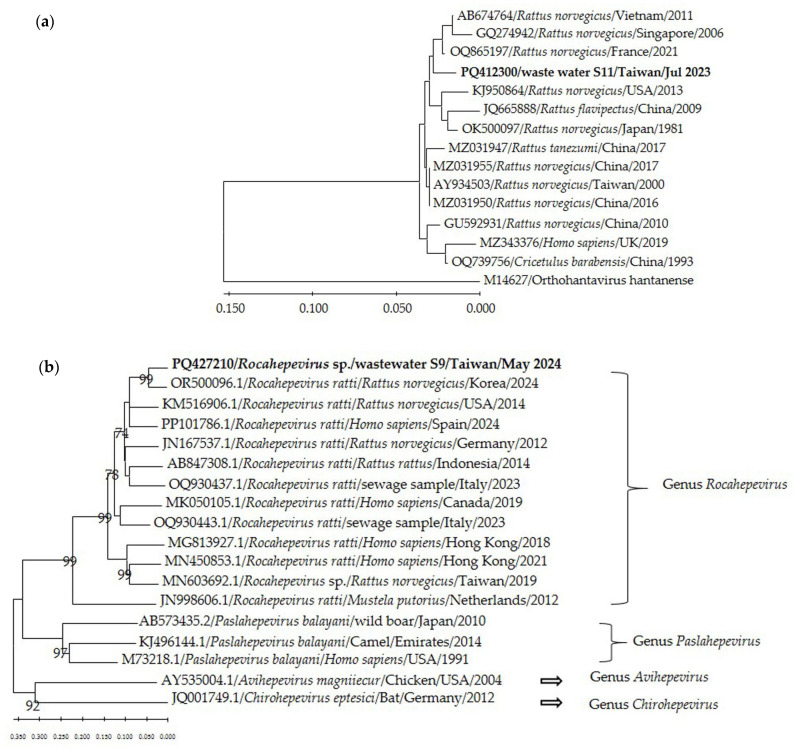
(**a**) The phylogenetic analysis of *Orthohantavirus seoulense* based on the partial nucleotide sequence of the M segment (160 nt). The evolutionary relationships were inferred by a neighbor-joining method with 1000 bootstrap replicates. (**b**) The phylogenetic analysis of hepeviruses based on the partial nucleotide sequence of ORF1 (279 nt). The evolutionary relationships were inferred by a neighbor-joining method with 1000 bootstrap replicates. Sequences obtained in the study were written in bold, and the sampling sites and time were indicated.

Of the 131 wastewater samples tested, hantavirus RNA was identified in one sample collected at Site 11 in July 2023 (Table 2). Phylogenetic analysis showed that the sequence was 98.7% similar to the isolate from *R. norvegicus* (GU592931) (Figure 3a). Five human cases were diagnosed during June 2023 to May 2024, but only one case occurred in the Taipei Region in June 2023.

### 3.4. Detection of HEV/RHEV in Wastewater and Human Cases with Hepatitis E

Only one wastewater sample collected at Site 9 in May 2024 contained HEV/RHEV RNA (Table 2). Phylogenetic analysis revealed that the sequence was most similar to the *Rocahepevirus* isolate derived from *R. norvegicus* in Korea (OR500096) (Figure 3b). Although five confirmed cases of HEV were diagnosed, no human case of RHEV was reported during the study period.

### 3.5. Detection of Rodent DNA in Wastewater

Rodent cDNA was found in 25 (25/131, 19.1%) samples. While Site 2, 3, and 10 yielded positive results most frequently (4 of 12 months, 33.3%), rodent cDNA was detectable at least once at each site except Site 4 during the 12-month study period. The positivity rate in May 2024 was 45.5% (5 of 11 sites), but all samples collected in August, September, and October in 2023 and February in 2024 were tested negative. There was no significant difference in the number of positive samples between seasons (*F* = 1.1, *p* = 0.416). Sequences of the amplicons of the partial ND2 gene fell into two groups, with similarities of 100% and 97.5% to the reference sequences (FQ230163 and FQ216572) from *R. norvegicus* in GenBank. The presence of rodent cDNA in wastewater coincided with the detection of pathogens in eight samples. Nevertheless, the number of positive samples for rodent cDNA was neither correlated with the positivity rates of tested rodent-borne pathogens in each month nor at each site or the number of patients (r = 0.08, 0.048, and −0.188; *p* = 0.98, 0.889, and 0.559, respectively).

## 4. Discussion

Wastewater monitoring has been proven as a useful tool for tracking epidemics in communities. In the study, we examined rodent-borne pathogens such as *Leptospira*, hantaviruses, and RHEV in wastewater collected along the Tamsui sewer system and found an overall positivity rate of 38.2% (50/131). While *Leptospira* was detected most often in the wastewater samples (48/131, 36.6%), RHEV and hantaviruses were found only once each during the 12-month study period. Meanwhile, Site 6 and 9 had the highest detection rate (8 of 12 months, 66.7%) of rodent-borne pathogens studied among all sampling sites.

*Leptospira* has been shown to be present commonly in urban environments. A study conducted in Sydney, Australia discovered pathogenic *Leptospira* in 35/75 water or soil samples in 20 parks [53]. In a Brazilian urban slum with high infection rates (37.8 per 1000 individuals per year) of leptospirosis, 36.1% (121/335) of sewage samples from the open sewer were positive for *Leptospira* DNA [54]. Despite the few numbers of cases and the developed urban environment with underground sewer systems, our study still detected a relatively high positivity rate for *Leptospira* (36.6%). In addition, cDNA was used for the detection of rodent-borne pathogens in the study for convenience. Given that RNA is less stable and susceptible to RNase degradation in wastewater, the positivity rate may be underestimated. Sequencing of the amplicons identified *L. interrogans* in our wastewater samples, with diversities in sequences. Moreover, different sequences were found in samples collected at different sites in the same month and at the same site in different months. For example, the amplicon sequences of samples collected at Site 9 (PQ456232) and Site 10 (PQ456221) in November 2023 shared 99.6% similarity; meanwhile, the amplicon sequences of samples collected at Site 9 in November 2023 (PQ456232) and February 2024 (PQ456225) were 99.6% similar. Although we were unable to access human cases, the change in pathogen sequences could also result from movement of rodents or interactions between rodent populations in the sewer system and those active in surrounding habitats. Nevertheless, the same sequence was detected in samples collected in different months and locations, suggesting a continuous transmission cycle of the pathogen in the region.

*Leptospira* is the most widespread zoonosis in the world, affecting not only developing countries but also urban cities in developed industrialized countries [55,56]. Rodents active in urban as well as suburban environments, such as *R. norvegicus* and *R. rattus*, play important roles in the transmission of *Leptospira* [57]. Contamination has been reported in sewage, sewage sludge, soil, and surface water, and leptospirosis is recognized as an occupational hazard for sewage workers, public cleansing workers, miners, and rice farmers [55,58,59]. In Taiwan, leptospirosis is a neglected disease, and research regarding its epidemiology is scarce. Our discovery of pathogenic *Leptospira* in wastewater highlighted the threat of epidemics. Outbreaks of leptospirosis are often associated with natural disasters accompanying heavy rains. As global climate change is expected to bring more frequent or intense extreme weather events, precautions should be taken against leptospirosis. Moreover, positive samples were collected along the sewer system including influent and effluent water at the wastewater treatment plant. Previous studies have observed that the relative proportion of *Legionella* and *Leptospira* increased while the relative abundance of other disease-associated bacteria reduced by the wastewater treatment process [60]. However, the detection rate decreased from 41.7% (5/12) at Site 10 to 18.2% (2/11) at Site 11, although the relative proportions of microorganisms were not measured in our work. *Leptospira* was not detectable in three samples after treatment. The improvement may result from the filtration and UV-disinfection procedure employed by the Tamsui Wastewater Treatment Plant for purification before discharging wastewater into the ocean.

Hantavirus RNA was identified only in a sample collected at Site 11, suggesting contamination within the wastewater treatment plant. Genetic sequence data showed that the detected virus was closest to the reference sequences from *R. norvegicus*, implying a rodent origin or a novel human pathogen. Further investigation into the virus exposure in the rodent reservoirs and workers around the wastewater treatment plant would give a better understanding of the epidemiology of hantaviruses. On the other hand, we detected RHEV in a wastewater sample despite a lack of reports of human cases in Taiwan. The finding may provide a warning sign of epidemics. Otherwise, phylogenetic analysis revealed that the sequence exhibited more similarity to those found in rodents than to reference sequences in Genbank isolated from patients. The same observation has been described previously. Studies in Italy, Spain, and Sweden have reported prevalence of 9.3%, 0.9%, and 86% for HEV and 90.7%, 94.3%, and 98% for RHEV, respectively, but all the sequences grouped in different genetic clusters with a geographic-related pattern, and none of them were genetically close to strains found in human cases [19,20,21]. Another study in Sweden identified five strains of HEV and two strains of RHEV in 7 of 11 effluents. Four of the HEV were similar to strains isolated from humans in 2014 and 2015, while the two RHEV strains may belong to a new genotype [22].

The rodent-borne pathogens detected in the study may result from infected individuals as well as direct contamination by synanthropic animals inhabiting the sewer system. We then examined wastewater for rodent genetic materials, and as expected, identified *R. norvegicus*, confirming rodent activities in the sewer system. Studies have demonstrated the complex ecology of urban rat populations, and trapping methods would affect the captures and thus inferences drawn from the collected rodents [57]. Analyzing wastewater could provide a noninvasive alternative for research on rodent ecology and vector epidemiology. Markers of sample contamination by feces or urine from animal reservoirs could help to distinguish human infections and animal-derived pathogens [9]. Combining the genetic sequence data and co-occurrence of rodent genetic material with hantavirus in the wastewater sample suggested that an animal origin rather than human infection was more likely.

Nevertheless, our study did not find significant correlations between the detection of rodent-borne pathogens and the numbers of confirmed human cases or rodent cDNA. Studies of the COVID-19 pandemic suggested that surveillance of SARS-CoV-2 in wastewater could outperform case numbers for tracking COVID-19 incidence dynamics when test positivity rates were high [61]. Compared with SARS-CoV-2, our results showed a relatively lower prevalence of 0.8 (1/131)–36.6% (48/131) for the rodent-borne pathogens. Similarly, a study in Spain noticed no correlation of clinical cases and the detection of HEV and RHEV in wastewater, although another report demonstrated that the sewage concentrations of hantavirus were consistent with the trends in the sentinel hospital percent positivity [20,62]. Rodents are main reservoirs for *Leptospira*, hantaviruses, and RHEV, and rodents active in the sewer system have been reported being infected by the pathogens [63]. The complicated interactions between pathogens, reservoir animals, and humans in zoonosis transmission should be taken into consideration. Even pathogens excreted by infected animal hosts were detectable in wastewater, probabilities of ingesting contaminated water would depend on other determinants, such as a flood, leakage in tape water pipelines, occupational exposure, etc. In this context, monitoring rodent-borne pathogens in wastewater could provide information about the prevalence of infections in animal hosts and thus zoonotic threat in the area [64].

The detection of *Leptospira*, hantaviruses, and RHEV in wastewater when human cases were absent could be influenced by strains of pathogens, especially for the hantaviruses and RHEV identified in the study, which were genetically close to strains isolated from animals. Whether these microorganisms would cause diseases in humans remains to be investigated. Further, the detected pathogens may come from zoonotic sources, such as companion animals, while dogs and cats have been identified as potential carriers of *Leptospira*, HEV, and RHEV [65,66,67]. Rodent movement along the sewers could also affect the detection of pathogens and rodent genetic materials. On the other hand, only symptomatic patients diagnosed by physicians are reported to the Taiwan CDC, whereas these rodent-borne diseases often present as subclinical or asymptomatic infections. Infected individuals not seeking medical help were overlooked, resulting in an underestimation in the number of patients.

Decay kinetics of pathogens in sewer systems would affect the accuracy of detection of pathogens in wastewater. Pathogens may have different decay characteristics within different physical and chemical conditions [68,69,70]. As survival of viral RNA through the sewage can be influenced by dilution, temperature variation, microbial adsorption and degradation, interaction with other metabolites, etc., bacterial pathogens may exhibit high decay rates in the sewer system or grow and thrive if the environments are rich in nutrients [71,72]. Taking *Leptospira* as an example, increases in pH were associated with increased concentrations of pathogenic *Leptospira* [73]. Pathogenic *Leptospira* can survive at water temperatures ranging between 25 and 30 °C [74]. The temperature, pH, and DO of samples positive for *Leptospira* ranged from 16.8 to 30.6 °C (24.9 ± 3.3 °C), 6.3 to 9.0 (8.0 ± 0.5), and 0.8 to 7.2 mg/L (2.9 ± 1.4 mg/L) in the study.

Three limitations for the current study should be mentioned. Limited by data granularity and personal information protection, we were unable to obtain the addresses of patients and performed analyses against sampling sites. Due to the molecular assays used in the study, we did not quantify the pathogen DNA/RNA and carry out the correlation analyses with case numbers and sampling sites. In addition, grab sampling provided an easy way for sampling through manholes, which were sometimes located in the middle of main roads and required the traffic to stop for sampling. Yet, this method may not be able to retain information-containing excreta only temporarily discharged into wastewater and reduced the sensitivity of the study. Since rodents are natural carriers of *Leptospira* and hantaviruses, infections are often chronical and subclinical and result in persistent pathogen shedding [75,76]. The transmission route of RHEV is still not clear [77]. Considering the relatively low number of cases caused by the target pathogens and availability of the workforce, sampling was performed once a month in the study.

Currently, most studies of wastewater-based epidemiology were conducted on samples collected from wastewater treatment plants [19,20,21,22,60,61,78]. In the study, we collected wastewater samples from manholes along a sewer system as well as influents and effluents at a wastewater treatment plant. Low concentrations of pathogens excreted into upstream sewage may not be detectable at wastewater treatment plants due to dilution, precipitation, sedimentation, and degradation. The strategy of sampling along sewer systems would enhance sensitivity and help to locate specific areas of risk. Notably, the results of certain sampling sites (sampling points) may not be good indicators of the main residential neighborhoods. For example, a sudden rise in viral load at a sampling site due to a gathering event does not represent the overall epidemic situation in the area. Sampling from specific sites may introduce confirmation bias when assessing a disease burden in a community [79].

Our study showed the presence of potential rodent-borne pathogens including *Leptospira*, hantaviruses, and RHEV in wastewater in Tamsui, a district of the Taipei Metropolitan Area. The findings implied that the rodent-borne diseases may be underdiagnosed and highlighted the need for increased awareness of the infections. Continuous surveillance of rodent-borne zoonoses and reservoir animals using wastewater samples would provide valuable epidemiological information in the community.

The risk of emergence and spread of novel zoonotic pathogens is multiplying amid global warming and frequent international travel. The World Health Organization therefore prioritized Disease X, a possible pandemic, to help in preparation for future outbreaks. Incorporating wastewater-based epidemiology into the One Health approach will greatly improve the efficiency of surveillance of emerging zoonotic infections and facilitate proactive planning for disease prevention and control.

## 5. Conclusions

We first identified potential rodent-borne pathogens including *Leptospira*, hantaviruses, and RHEV in wastewater in Taiwan. Phylogenetic analysis revealed that the *Leptospira* spp. were similar to strains found in animals and patients, while the *Orthohantavirus* and RHEV were close to isolates from rodents. The discrepancy between the prevalence of pathogen DNA/RNA and human cases may suggest undetected infections in reservoir hosts or humans. Wastewater-based epidemiology could be applied as a One Health approach to provide a comprehensive overview of emerging zoonotic pathogens in the community.

## Figures and Tables

**Table 1:** Quality indicators in each month (mean ± SD) for wastewater samples collected in Tamsui, Taiwan during June 2023 to May 2024.

	2023							2024				
	Jun	Jul	Aug	Sep	Oct	Nov	Dec	Jan	Feb	Mar	Apr	May
Temperature (°C)	25.8 ± 0.5	28.5 ± 1.3	28.0 ± 0.8	27.8 ± 2.1	25.6 ± 1.1	23.3 ± 2.1	24.1 ± 1.4	20.7 ± 1.0	21.8 ± 1.8	21.0 ± 2.4	27.7 ± 2.7	26.1 ± 2.5
pH	8.0 ± 0.4	7.8 ± 0.6	8.2 ± 0.4	8.2 ± 0.5	7.9 ± 0.3	8.1 ± 0.4	7.5 ± 0.3	8.3 ± 0.8	8.0 ± 0.7	7.9 ± 0.7	7.8 ± 0.6	7.9 ± 0.7
Dissolved oxygen (mg/L)	2.2 ± 0.9	2.6 ± 1.2	2.5 ± 1.2	2.4 ± 1.1	2.4 ± 1.1	2.4 ± 1.2	2.5 ± 1.3	2.9 ± 1.1	3.3 ± 0.9	3.9 ± 1.3	4.8 ± 2.4	3.4 ± 1.7
Biochemical oxygen demand (BOD) (mg/L)	80.5 ± 56.1	54.5 ± 23.4	52.7 ± 32.5	58.4 ± 43.7	175.7 ± 39.0	95.2 ± 41.0	58.8 ± 26.7	80.1 ± 57.3	51.0 ± 31.2	45.8 ± 16.7	57.6 ± 20.9	83.8 ± 32.0
Chemical oxygen demand (COD) (mg/L)	235.3 ± 161.3	118.8 ± 49.8	118.7 ± 55.1	138.9 ± 69.2	122.6 ± 56.8	167.0 ± 84.7	129.8 ± 48.0	167.6 ± 156.4	133.7 ± 44.0	182.3 ± 67.1	141.3 ± 48.0	225.2 ± 58.4
NH3-N (mg/L)	57.3 ± 10.9	30.7 ± 11.9	27.2 ± 14.1	31.8 ± 10.6	32.4 ± 10.3	42.9 ± 13.2	37.1 ± 15.7	46.2 ± 15.2	37.7 ± 17.0	36.5 ± 14.0	30.3 ± 15.4	44.5 ± 14.6
Total nitrogen (mg/L)	56.8 ± 10.9	37.9 ± 10.6	40.0 ± 11.9	42.9 ± 12.0	63.3 ± 16.4	49.1 ± 10.6	47.5 ± 11.6	54.9 ± 15.4	50.7 ± 12.8	57.8 ± 11.8	52.0 ± 13.1	50.0 ± 14.3
Total phosphate (mg/L)	0.0 ± 0.0	3.1 ± 0.7	3.2 ± 0.8	3.4 ± 1.2	3.2 ± 0.7	4.1 ± 1.4	4.1 ± 0.8	3.8 ± 1.0	3.5 ± 0.8	3.7 ± 1.3	3.0 ± 1.1	4.8 ± 0.8

**Table 2:** Sampling sites at which genetic materials of rodent-borne pathogens and rodents were detected in wastewater collected in Tamsui, Taiwan during June 2023 to May 2024.

	2023							2024				
	Jun * (%)	Jul (%)	Aug (%)	Sep (%)	Oct (%)	Nov (%)	Dec (%)	Jan (%)	Feb (%)	Mar (%)	Apr (%)	May (%)
*Leptospira*	2, 6 (20)	4, 6 (18.2)	3, 4, 5, 9, 10, 11 (54.5)	1, 2, 3, 6, 7 (45.5)	2, 3, 6, 7 (36.4)	5, 6, 9, 10 (36.4)	1, 6, 9 (27.3)	1, 3, 7, 9, 10 (45.5)	4, 6, 9 (27.3)	2, 3, 5, 7, 9, 10 (54.5)	1, 9, 10, 11 (36.4)	4, 5, 6, 8 (36.4)
Hantaviruses	ND (0)	11 (9.1)	ND (0)	ND (0)	ND (0)	ND (0)	ND (0)	ND (0)	ND (0)	ND (0)	ND (0)	ND (0)
HEV/RHEV	ND (0)	ND (0)	ND (0)	ND (0)	ND (0)	ND (0)	ND (0)	ND (0)	ND (0)	ND (0)	ND (0)	9 (9.1)
Rodent	3, 7, 10 (30)	2, 3, 8, 10, 11 (45.5)	ND (0)	ND (0)	ND (0)	2, 5 (18.2)	10 (9.1)	3, 5, 9, 10 (36.4)	ND (0)	1, 2, 6, 11 (36.4)	7 (9.1)	1, 2, 3, 6, 7 (45.5)

ND: not detected. * Only samples from Site 1 to 10 were collected in January 2023.

## Data Availability

The original contributions presented in this study can be found in the context. The obtained sequences were openly available in GenBank including the accession numbers of PQ456223, PQ456224, PQ456221, PQ456229, PQ456227, PQ456226, PQ456225, PQ456222, PQ456228, PQ456232, PQ456231, PQ456233, and PQ456230 for *Leptospira*; PQ412300 for hantavirus; and PQ427210 for RHEV.

## Supplementary Materials

The following supporting information can be downloaded at: https://www.mdpi.com/article/10.3390/tropicalmed9110282/s1, Table S1: Primers used in the study.

## References

[1] Hovi T., Shulman L.M., van der Avoort H., Deshpande J., Roivainen M., DE Gourville E.M. (2012). Role of environmental poliovirus surveillance in global polio eradication and beyond. Epidemiol. Infect..

[2] Sinclair R.G., Choi C.Y., Riley M.R., Gerba C.P. (2008). Pathogen surveillance through monitoring of sewer systems. Adv. Appl. Microbiol..

[3] Fernandez-Cassi X., Timoneda N., Martínez-Puchol S., Rusiñol M., Rodriguez-Manzano J., Figuerola N., Bofill-Mas S., Abril J.F., Girones R. (2018). Metagenomics for the study of viruses in urban sewage as a tool for public health surveillance. Sci. Total Environ..

[4] Graham K.E., Loeb S.K., Wolfe M.K., Catoe D., Sinnott-Armstrong N., Kim S., Yamahara K.M., Sassoubre L.M., Mendoza Grijalva L.M., Roldan-Hernandez L. (2021). SARS-CoV-2 RNA in wastewater settled solids is associated with COVID-19 cases in a large urban sewershed. Environ. Sci. Technol..

[5] Medema G., Heijnen L., Elsinga G., Italiaander R., Brouwer A. (2020). Presence of SARS-Coronavirus-2 RNA in sewage and correlation with reported COVID-19 prevalence in the early stage of the epidemic in The Netherlands. Environ. Sci. Technol. Lett..

[6] Peccia J., Zulli A., Brackney D.E., Grubaugh N.D., Kaplan E.H., Casanovas-Massana A., Ko A.I., Malik A.A., Wang D., Wang M. (2020). Measurement of SARS-CoV-2 RNA in wastewater tracks community infection dynamics. Nat. Biotechnol..

[7] Jahn K., Dreifuss D., Topolsky I., Kull A., Ganesanandamoorthy P., Fernandez-Cassi X., Bänziger C., Devaux A.J., Stachler E., Caduff L. (2022). Early detection and surveillance of SARS-CoV-2 genomic variants in wastewater using COJAC. Nat. Microbiol..

[8] Zoonotic Disease: Emerging Public Health Threats in the Region. https://www.emro.who.int/fr/about-who/rc61/zoonotic-diseases.html.

[9] Grassly N.C., Shaw A.G., Owusu M. (2024). Global wastewater surveillance for pathogens with pandemic potential: Opportunities and challenges. Lancet Microbe.

[10] Hill R., Stentiford G.D., Walker D.I., Baker-Austin C., Ward G., Maskrey B.H., van Aerle R., Verner-Jeffreys D., Peeler E., Bass D. (2024). Realising a global One Health disease surveillance approach: Insights from wastewater and beyond. Nat. Commun..

[11] Adams C., Kirby A.E., Bias M., Riser A., Wong K.K., Mercante J.W., Reese H. (2024). Detecting mpox cases through wastewater surveillance—United States, August 2022-May 2023. MMWR Morb. Mortal. Wkly. Rep..

[12] Bagutti C., Alt Hug M., Heim P., Ilg Hampe E., Hübner P., Julian T.R., Koch K.N., Grosheintz K., Kraus M., Schaubhut C. (2024). Association between the number of symptomatic mpox cases and the detection of mpox virus DNA in wastewater in Switzerland: An observational surveillance study. Swiss Med. Wkly..

[13] Xu J., Liu C., Zhang Q., Zhu H., Cui F., Zhao Z., Song M., Zhou B., Zhang Y., Hu P. (2024). The first detection of mpox virus DNA from wastewater in China. Sci. Total Environ..

[14] Wolfe M.K., Yu A.T., Duong D., Rane M.S., Hughes B., Chan-Herur V., Donnelly M., Chai S., White B.J., Vugia D.J. (2023). Use of wastewater for mpox outbreak surveillance in California. N. Engl. J. Med..

[15] Foulkes D., Kittner A., Korban C., Anderson K., DeJonge P.M., Faherty E.A.G., Kerins J.L., Poretsky R., Pierce M., Atwater R. (2024). Using wastewater surveillance for mpox as a complement to traditional case-based reporting—Chicago, March-June 2023. Environ. Int..

[16] Mejia E.M., Hizon N.A., Dueck C.E., Lidder R., Daigle J., Wonitowy Q., Medina N.G., Mohammed U.P., Cox G.W., Safronetz D. (2024). Detection of mpox virus in wastewater provides forewarning of clinical cases in Canadian cities. Sci. Total Environ..

[17] Honein M.A., Olsen S.J., Jernigan D.B., Daskalakis D.C. (2024). Challenges and opportunities for wastewater monitoring of influenza viruses during the multistate outbreak of highly pathogenic avian influenza A(H5N1) virus in dairy cattle and poultry. Am. J. Public. Health.

[18] Rouba A., Ansmant T., Chaqroun A., Challant J., Josse T., Schvoerer E., Gantzer C., Bertrand I., Hartard C. (2024). First detection of Hepatitis E virus (*Rocahepevirus ratti*) in French urban wastewater: Potential implications for human contamination. Sci. Total Environ..

[19] Palombieri A., Di Profio F., Sarchese V., Fruci P., Suffredini E., Martella V., Veneri C., Bonanno Ferraro G., Mancini P., La Rosa G. (2023). Surveillance for rat hepatitis E in wastewater networks, Italy. Microbiol. Spectr..

[20] Casares-Jimenez M., Garcia-Garcia T., Suárez-Cárdenas J.M., Perez-Jimenez A.B., Martín M.A., Caballero-Gómez J., Michán C., Corona-Mata D., Risalde M.A., Perez-Valero I. (2024). Correlation of hepatitis E and rat hepatitis E viruses urban wastewater monitoring and clinical cases. Sci. Total Environ..

[21] Churqui M.P., Ghaleb M., Tunovic T., Frankal M., Enache L., Nyström K., Lagging M., Wang H. (2024). High prevalence of hepatitis E and rat hepatitis E viruses in wastewater in Gothenburg, Sweden. One Health.

[22] Wang H., Neyvaldt J., Enache L., Sikora P., Mattsson A., Johansson A., Lindh M., Bergstedt O., Norder H. (2020). Variations among viruses in influent water and effluent water at a wastewater plant over one year as assessed by quantitative PCR and metagenomics. Appl. Environ. Microbiol..

[23] Wong J.C.C., Tay M., Hapuarachchi H.C., Lee B., Yeo G., Maliki D., Lee W., Mohamed Suhaimi N.A., Chio K., Tan W.C.H. (2024). Case report: Zika surveillance complemented with wastewater and mosquito testing. EBioMedicine.

[24] Monteiro S., Pimenta R., Nunes F., Cunha M.V., Santos R. (2024). Detection of dengue virus and chikungunya virus in wastewater in Portugal: An exploratory surveillance study. Lancet Microbe.

[25] The Taiwan National Infectious Disease Statistics System (NIDSS). https://nidss.cdc.gov.tw/Home/Index.

[26] Chou Y.L., Chen C.S., Liu C.C. (2008). Leptospirosis in Taiwan, 2001–2006. Emerg. Infect. Dis..

[27] Su H.P., Chan T.C., Chang C.C. (2011). Typhoon-related leptospirosis and melioidosis, Taiwan, 2009. Emerg. Infect. Dis..

[28] Wu Y.W., Hsu E.L., Lin T.H., Huang J.H., Chang S.F., Pai H.H. (2007). Seaport as a source of hantavirus: A study on isolated isles. Int. J. Environ. Health Res..

[29] Su S.W., Wu Y.C., Chang S.F., Ho L.L., Su C.F. (2015). Epidemiological investigation of hantavirus in rodents at international ports in Taiwan, 2010–2013. Taiwan Epidemiol. Bull..

[30] Lee Y.H., Chang S.F., Wang H.C., Hsieh J.W., Lin M.C., Yang S.Y. (2012). Seroepidemiological investigation on hantavirus prevalence in rodent population at international ports in Taiwan, 2007–2009. Taiwan Epidemiol. Bull..

[31] Hsieh J.W., Wang J.T., Huang T.M., Chen C.H. (2008). Epidemiology investigation of rodents as vectors for the hantavirus in Taiwan’s harbor areas. Taiwan. Epidemiol. Bull..

[32] Kamar N., Izopet J., Pavio N., Aggarwal R., Labrique A., Wedemeyer H., Dalton H.R. (2017). Hepatitis E virus infection. Nat. Rev. Dis. Primers.

[33] Hepatitis E Fact Sheet. https://www.who.int/en/news-room/fact-sheets/detail/hepatitis-e.

[34] Wang C.H. (2001). Hepatitis E virus infection in Taiwan: Prevalence of neutralizing anti-HEVne positive serum. Taiwan. Epidemiol. Bull..

[35] Rodriguez C., Marchand S., Sessa A., Cappy P., Pawlotsky J.M. (2023). Orthohepevirus C hepatitis, an underdiagnosed disease?. J. Hepatol..

[36] Wang B., Harms D., Yang X.L., Bock C.T. (2020). Orthohepevirus C: An expanding species of emerging hepatitis E virus variants. Pathogens.

[37] Robinson S.J., Borlang J., Himsworth C.G., Pearl D.L., Weese J.S., Dibernardo A., Osiowy C., Nasheri N., Jardine C.M. (2023). Rat hepatitis E virus in Norway rats, Ontario, Canada, 2018–2021. Emerg. Infect. Dis..

[38] Sridhar S., Yip C.C.Y., Wu S., Cai J., Zhang A.J., Leung K.H., Chung T.W.H., Chan J.F.W., Chan W.M., Teng J.L.L. (2018). Rat hepatitis E virus as cause of persistent hepatitis after liver transplant. Emerg. Infect. Dis..

[39] Sridhar S., Yip C.C., Wu S., Chew N.F., Leung K.H., Chan J.F., Zhao P.S., Chan W.M., Poon R.W., Tsoi H.W. (2021). Transmission of rat hepatitis E virus infection to humans in Hong Kong: A clinical and epidemiological analysis. Hepatology.

[40] Sridhar S., Yip C.C.Y., Lo K.H.Y., Wu S., Situ J., Chew N.F.S., Leung K.H., Chan H.S.Y., Wong S.C.Y., Leung A.W.S. (2022). Hepatitis E virus species c infection in humans, Hong Kong. Clin. Infect. Dis..

[41] Andonov A., Robbins M., Borlang J., Cao J., Hatchette T., Stueck A., Deschambault Y., Murnaghan K., Varga J., Johnston L. (2019). Rat hepatitis E virus linked to severe acute hepatitis in an immunocompetent patient. J. Infect. Dis..

[42] Rivero-Juarez A., Frias M., Perez A.B., Pineda J.A., Reina G., Fuentes-Lopez A., Freyre-Carrillo C., Ramirez-Arellano E., Alados J.C., Rivero A. (2022). Orthohepevirus C infection as an emerging cause of acute hepatitis in Spain: First report in Europe. J. Hepatol..

[43] Takahashi M., Kunita S., Kawakami M., Kadosaka T., Fujita H., Takada N., Miyake M., Kobayashi T., Ohnishi H., Nagashima S. (2022). First detection and characterization of rat hepatitis E Virus (HEV-C1) in Japan. Virus Res..

[44] Chiang P.S., Huang W.L., Chung H.H., Yang J.Y., Teng H.J. (2022). The first documented detection of the hepatitis E virus in rats in Taiwan. Taiwan. Epidemiol. Bull..

[45] de Abreu Fonseca C., Teixeira de Freitas V.L., Caló Romero E., Spinosa C., Arroyo Sanches M.C., da Silva M.V., Shikanai-Yasuda M.A. (2006). Polymerase chain reaction in comparison with serological tests for early diagnosis of human leptospirosis. Trop Med. Int. Health..

[46] Chin C., Chiueh T.S., Yang W.C., Yang T.H., Shih C.M., Lin H.T., Lin K.C., Lien J.C., Tsai T.F., Ruo S.L. (2000). Hantavirus infection in Taiwan: The experience of a geographically unique area. J. Med. Virol..

[47] Drexler J.F., Seelen A., Corman V.M., Fumie Tateno A., Cottontail V., Melim Zerbinati R., Gloza-Rausch F., Klose S.M., Adu-Sarkodie Y., Oppong S.K. (2012). Bats worldwide carry hepatitis E virus-related viruses that form a putative novel genus within the family Hepeviridae. J. Virol..

[48] Bachoon D.S., Redhead A.S.Z., Mead A.J. (2024). Mitochondrial DNA marker: A PCR approach for tracking rat (*Rattus rattus* and *Rattus norvegicus*) fecal pollution in surface water systems. Sci. Total. Environ..

[49] Sanger F., Coulson A.R. (1975). A rapid method for determining sequences in DNA by primed synthesis with DNA polymerase. J. Mol. Biol..

[50] Swindell S.R., Plasterer T.N., Swindell S.R. (1997). SEQMAN. Sequence Data Analysis Guidebook. Methods In Molecular Medicine™.

[51] Thompson J.D., Higgins D.G., Gibson T.J. (1994). CLUSTAL W: Improving the sensitivity of progressive multiple sequence alignment through sequence weighting, position-specific gap penalties and weight matrix choice. Nucleic Acids Res..

[52] Tamura K., Stecher G., Kumar S. (2021). MEGA11: Molecular Evolutionary Genetics Analysis Version 11. Mol. Biol. Evol..

[53] Lu X., Westman M.E., Mizzi R., Griebsch C., Norris J.M., Jenkins C., Ward M.P. (2024). Are pathogenic *Leptospira* species ubiquitous in urban recreational parks in Sydney, Australia?. Trop. Med. Infect. Dis..

[54] Casanovas-Massana A., Costa F., Riediger I.N., Cunha M., de Oliveira D., Mota D.C., Sousa E., Querino V.A., Nery N., Reis M.G. (2018). Spatial and temporal dynamics of pathogenic *Leptospira* in surface waters from the urban slum environment. Water Res..

[55] Levett P.N. (2001). Leptospirosis. Clin. Microbiol. Rev..

[56] Socolovschi C., Angelakis E., Renvoisé A., Fournier P.E., Marié J.L., Davoust B., Stein A., Raoult D. (2011). Strikes, flooding, rats, and leptospirosis in Marseille, France. Int. J. Infect. Dis..

[57] Himsworth C.G., Jardine C.M., Parsons K.L., Feng A.Y., Patrick D.M. (2014). The characteristics of wild rat (*Rattus* spp.) populations from an inner-city neighborhood with a focus on factors critical to the understanding of rat-associated zoonoses. PLoS ONE.

[58] Jones P.W., Rennison L.M., Matthews P.R., Collins P., Brown A. (1981). The occurrence and significance to animal health of *Leptospira, Mycobacterium*, *Escherichia coli*, *Brucella abortus* and *Bacillus anthracis* in sewage and sewage sludges. J. Hyg..

[59] Boey K., Shiokawa K., Rajeev S. (2019). *Leptospira* infection in rats: A literature review of global prevalence and distribution. PLoS Negl. Trop. Dis..

[60] Numberger D., Ganzert L., Zoccarato L., Mühldorfer K., Sauer S., Grossart H.P., Greenwood A.D. (2019). Characterization of bacterial communities in wastewater with enhanced taxonomic resolution by full-length 16S rRNA sequencing. Sci. Rep..

[61] Fernandez-Cassi X., Scheidegger A., Bänziger C., Cariti F., Tuñas Corzon A., Ganesanandamoorthy P., Lemaitre J.C., Ort C., Julian T.R., Kohn T. (2021). Wastewater monitoring outperforms case numbers as a tool to track COVID-19 incidence dynamics when test positivity rates are high. Water Res..

[62] Li H., He F., Lv Z., Yi L., Zhang Z., Li H., Fu S. (2024). Tailored wastewater surveillance framework uncovered the epidemics of key pathogens in a Northwestern city of China. Sci. Total Environ..

[63] Krøjgaard L.H., Villumsen S., Markussen M.D., Jensen J.S., Leirs H., Heiberg A.C. (2009). High prevalence of *Leptospira* spp. in sewer rats (*Rattus norvegicus*). Epidemiol. Infect..

[64] dos Santos D.R., de Paula V.S., de Oliveira J.M., Marchevsky R.S., Pinto M.A. (2011). Hepatitis E virus in swine and effluent samples from slaughterhouses in Brazil. Vet. Microbiol..

[65] Zaidi S., Bouam A., Bessas A., Hezil D., Ghaoui H., Ait-Oudhia K., Drancourt M., Bitam I. (2018). Urinary shedding of pathogenic *Leptospira* in stray dogs and cats, Algiers: A prospective study. PLoS ONE.

[66] Caballero-Gómez J., Rivero-Juarez A., Jurado-Tarifa E., Jiménez-Martín D., Jiménez-Ruiz E., Castro-Scholten S., Ulrich R.G., López-López P., Rivero A., García-Bocanegra I. (2022). Serological and molecular survey of hepatitis E virus in cats and dogs in Spain. Transbound. Emerg. Dis..

[67] Shun E.H., Situ J., Tsoi J.Y., Wu S., Cai J., Lo K.H., Chew N.F., Li Z., Poon R.W., Teng J.L. (2024). Rat hepatitis E virus (*Rocahepevirus ratti*) exposure in cats and dogs, Hong Kong. Emerg. Microbes Infect..

[68] Li J., Ahmed W., Metcalfe S., Smith W.J.M., Choi P.M., Jackson G., Cen X., Zheng M., Simpson S.L., Thomas K.V. (2023). Impact of sewer biofilms on fate of SARS-CoV-2 RNA and wastewater surveillance. Nat. Water.

[69] Zhang S., Sharma E., Tiwari A., Chen Y., Sherchan S.P., Gao S., Zhou X., Shi J., Jiang G. (2023). The reduction of SARS-CoV-2 RNA concentration in the presence of sewer biofilms. Water.

[70] McLellan S.L., Eren A.M. (2014). Discovering new indicators of fecal pollution. Trends Microbiol..

[71] Parra-Arroyo L., Martínez-Ruiz M., Lucero S., Oyervides-Muñoz M.A., Wilkinson M., Melchor-Martínez E.M., Araújo R.G., Coronado-Apodaca K.G., Bedran H.V., Buitrón G. (2023). Degradation of viral RNA in wastewater complex matrix models and other standards for wastewater-based epidemiology: A review. TrAC Trends Anal. Chem..

[72] Tiwari A., Radu E., Kreuzinger N., Ahmed W., Pitkänen T. (2024). Key considerations for pathogen surveillance in wastewater. Sci. Total Environ..

[73] de Oliveira D., Airam Querino V., Sara Lee Y., Cunha M., Nery Jr. N., Wessels Perelo L., Rossi Alva J.C., Ko. A.I., Reis M.G., Casanovas-Massana A. (2020). Relationship between physicochemical characteristics and pathogenic *Leptospira* in urban slum waters. Trop. Med. Infect. Dis..

[74] Parker J., Walker M. (2011). Survival of a pathogenic *Leptospira* serovar in response to combined in vitro pH and temperature stresses. Vet. Microbiol..

[75] Leptospirosis Fact Sheet. https://iris.who.int/bitstream/handle/10665/205437/B4221.pdf.

[76] Voutilainen L., Sironen T., Tonteri E., Bäck A.T., Razzauti M., Karlsson M., Wahlström M., Niemimaa J., Henttonen H., Lundkvist Å. (2015). Life-long shedding of Puumala hantavirus in wild bank voles (*Myodes glareolus*). J. Gen. Virol..

[77] Li T.C., Yoshizaki S., Ami Y., Suzaki Y., Johne R., Wakita T. (2017). No Evidence of rat hepatitis E virus excretion in urine samples of rats. Jpn. J. Infect. Dis..

[78] Gonzalez R., Curtis K., Bivins A., Bibby K., Weir M.H., Yetka K., Thompson H., Keeling D., Mitchell J., Gonzalez D. (2020). COVID-19 surveillance in Southeastern Virginia using wastewater-based epidemiology. Water Res..

[79] Brighton K., Fisch S., Wu H., Vigil K., Aw T.G. (2024). Targeted community wastewater surveillance for SARS-CoV-2 and Mpox virus during a festival mass-gathering event. Sci. Total Environ..

