# Supplementary material for: Surveillance of Emerging Rodent-Borne Pathogens in Wastewater in Taiwan: A One Health Approach
Source: Trop Med Infect Dis. 2024 Nov 18;9(11):282. doi: 10.3390/tropicalmed9110282 (PMC11598759; doi:10.3390/tropicalmed9110282)
Supplement: Supplementary file 1 [file tropicalmed-09-00282-s001.zip › tropicalmed-3273564-supplementary.pdf]

Table S1. Primers used in the study

Host	Sequence (5′-3′)	Expected size	Reference
Leptospira	G2F: GGAAAACAAATGGTCGGAAG	285	[1]
	G1R: CTGAATCGCTGTATAAAAGT		
hantaviruses	HTN-SEO 1F: GATATGAATGATTG (T/C) TTTGT	311	[2]
	HTN-SEO 1R: CCATCAGGGTCT (T/C) TCCA		
	2F: TGTATAATTGGGAC (T/A) GTATCTAA	161	
	2R: GCAAAGTTACATTT (T/C) TTCCT		
HEV/RHEV	HEV-cs: TCGCGCATCACMTTYTTCCARAA	469–472	[3]
	HEV-cas: GCCATGTTCCAGACDGTRTTCCA		
	HEV-csn: TGTGCTCTGTTTGGCCCNTGGTTYCG	331–334	
	HEV-casn: CCAGGCTCACCRGARTGYTTCTTCCA		
Rat	RatMt-F: GGACTAGCCCCCTTCCACTA	180	[4]
	RatMt-R: GGCGCCAACAAAGACTGATG		

Reference

1. de Abreu Fonseca, C.; Teixeira de Freitas, V.L.; Caló Romero, E.; Spinosa, C.; Arroyo Sanches, M.C.; da Silva, M.V.; Shikanai-Yasuda, M.A. Polymerase chain reaction in comparison with serological tests for early diagnosis of human leptospirosis. *Trop Med Int Health* **2006**, *11(11)*, 1699–1707. https://doi.org/10.1111/j.1365-3156.2006.01727.x.

2. Chin, C.; Chiueh, T.S.; Yang, W.C.; Yang, T.H; Shih, C.M.; Lin, H.T.; Lin, K.C.; Lien, J.C.; Tsai, T.F.; Ruo, S.L.; Nichol, S.T.; Ksiazek, T.G.; Rollin, P.E.; Peters, C.J.; Wu, T.N.; Shen, C.Y. Hantavirus infection in Taiwan: the experience of a geographically unique area. *J Med Virol* **2000**, *60(2)*, 237–247. https://doi.org/10.1002/(sici)1096-9071(200002)60:2<237::aid-jmv21>3.0.co;2-b.

3. Drexler, J.F.; Seelen, A.; Corman, V.M.; Fumie Tateno, A.; Cottontail, V.; Melim Zerbinati, R.; Gloza-Rausch, F.; Klose, S.M.; Adu-Sarkodie, Y.; Oppong, S.K.; Kalko, E.K.; Osterman, A.; Rasche, A.; Adam, A.; Müller, M.A.; Ulrich, R.G.; Leroy, E.M.; Lukashev, A.N.; Drosten, C. Bats worldwide carry hepatitis E virus-related viruses that form a putative novel genus within the family Hepeviridae. *J Virol* **2012**, *86(17)*, 9134–9147. https://doi.org/10.1128/JVI.00800-12.

4. Bachoon, D.S.; Redhead, A.S.Z.; Mead, A.J. Mitochondrial DNA marker: A PCR approach for tracking rat (*Rattus rattus* and *Rattus norvegicus*) fecal pollution in surface water systems. *Sci Total Environ* **2024**, *921*, 171164. https://doi.org/10.1016/j.scitotenv.2024.171164.
